# Effect of external cues on clock-driven protection from influenza A infection

**DOI:** 10.1172/JCI193133

**Published:** 2025-11-17

**Authors:** Oindrila Paul, Thomas G. Brooks, Alisha Shetty, Y. Jane Choi, Martina Towers, Lora J. Assi, James P. Garifallou, Kaitlyn Forrest, Alecia Cameron, Amita Sehgal, Gregory Grant, Shaon Sengupta

**Affiliations:** 1 Children’s Hospital of Philadelphia, Philadelphia, Pennsylvania, USA.; 2 Institute of Translational Medicine and Therapeutics, University of Pennsylvania, Philadelphia, Pennsylvania, USA.; 3 Department of Neuroscience,; 4 Department of Genetics, and; 5 Department of Pediatrics, University of Pennsylvania Perelman School of Medicine, Philadelphia, Pennsylvania, USA.

**Keywords:** Immunology, Inflammation, Pulmonology, Influenza, Innate immunity, T cells

## Abstract

Influenza and other respiratory viral pathogens remain leading causes of mortality and morbidity. Circadian rhythms play a critical role in regulating immune responses and can confer temporal protection from influenza infection. Here, we investigated whether this protection requires rhythmic function after the initial infection by manipulating environmental cycles. We found that disrupting environmental lighting cues within a critical window of vulnerability abrogated the time-of-day-specific protection. This poor outcome was mediated by a dysregulated immune response, as evidenced by the accumulation of inflammatory monocytes and CD8^+^ T cells in the lungs and a transcriptomic profile indicative of an exaggerated inflammation. Disruption of the light cycle did not affect outcomes in a clock mutant, indicating that it acts through the host’s circadian clock. Importantly, rhythmic meal timing mitigated the adverse effects of disrupted light cycles, supporting the idea that external cues acting through different body clocks can compensate for one another. Together, these findings underscore the critical interplay between environmental timing cues and endogenous circadian rhythms in determining influenza outcomes and offer translational insight into optimizing care for critically ill patients with respiratory viral infections.

## Abstract 2

Our study shows that disruptions in daily light cycles after infection, worsen flu outcomes, but timed meals can restore protection—highlighting how circadian rhythms shape immune defense

## Graphical abstract

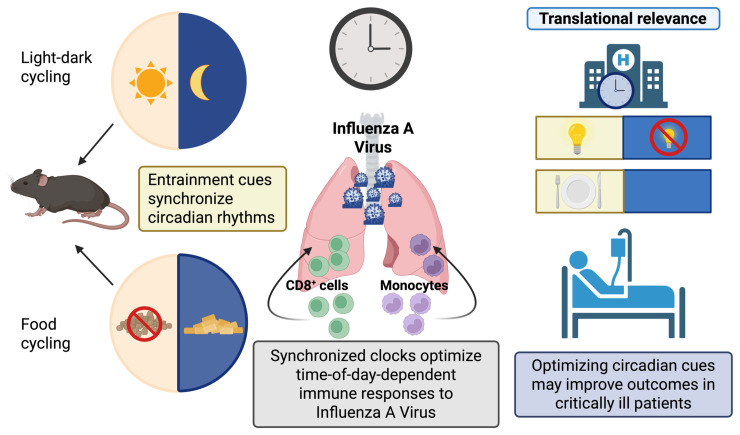

## Introduction

Circadian rhythms provide an anticipatory protective mechanism in the face of environmental challenges, including the threat of infections. In mammals, the master pacemaker resides in the suprachiasmatic nucleus (SCN) (1). However, almost every cell has its cell-intrinsic clock or molecular clock. Rhythmic external cues, or zeitgebers, synchronize central and peripheral clocks, ensuring optimal organismal function (2). Light is the most important zeitgeber for the SCN, which then synchronizes other body clocks; however, other organs and cells are more sensitive to entrainment to organ-specific cues. Acute or chronic disruptions of entraining cues are well known to disrupt synchronization between the central and peripheral clocks, with their harmful effects best documented in chronic states such as shift work (3, 4). However, the impact of external entrainment in an acute context, mainly as it affects host defense, is less well understood.

Previously, we showed that the circadian clock confers time-of-day-specific protection from influenza infection. Mice infected at dawn had a 3-fold better survival rate than mice infected at dusk. This advantage was lost in mice with a genetically disrupted circadian clock (*Bmal1^–/–^*) (5). However, mechanisms underlying this time-of-day effect remain unclear — for instance, is cycling physiology required only at the time of infection, or does it need to be sustained for a period after infection? The extended time frame (8–10 days) from infection to peak mortality in the influenza model renders it well-suited to address this issue. This study examines how zeitgebers influence time-of-day-specific protection in clock-intact hosts. We hypothesize that sustained exposure to rhythmic external cues is necessary to maintain this protection after initial infection. Using light-dark cycles and meal timing as model zeitgebers, we assessed their effect on time-of-day variations in influenza infection outcomes.

## Results

### Time-of-day-specific protection from influenza infection is lost after constant light exposure.

**Figure 1:**
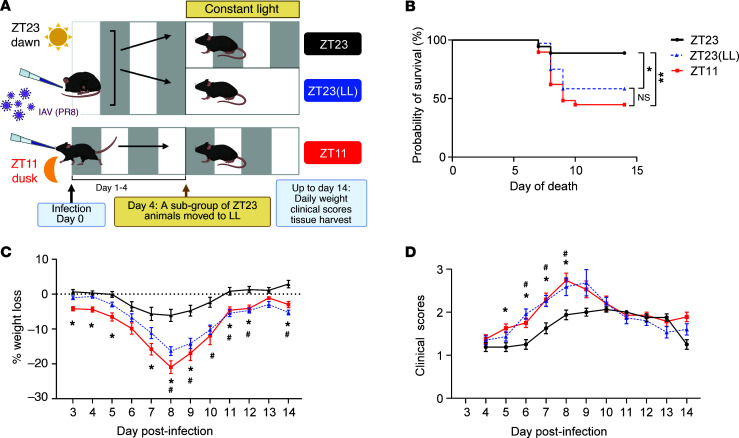
Constant light exposure abrogated the specific time-of-day protection following influenza A infection. (**A**) Experimental model. (**B**) Survival (**P < 0.01, log-rank test; *P < 0.05, log-rank test from 3 independent experiments). (**C**) Weight loss trajectory (*P < 0.001, ANOVA for repeated measures; ^#^*P* < 0.001, ANOVA for repeated measures). (**D**) Average clinical score as a representation of disease progression (*P < 0.001, ANOVA for repeated measures; ^#^*P* < 0.001, ANOVA for repeated measures) following IAV infection (*n* = 16–36 per group). All data were pooled from 3–5 independent experiments. ZT23 vs. ZT11 comparisons are indicated with an *; ZT23 vs. ZT23(LL) comparisons are indicated with a #.

We have previously shown that mice infected at dawn had 3-fold higher survival compared with mice infected at dusk (5). We hypothesized that abrogation of cyclical photic cues caused by constant light exposure would abolish the time-of-day-specific protection from influenza A virus (IAV) driven by the endogenous circadian clock. By circadian terminology, ZT0 refers to the time when the lights turn on, and ZT12 is when the lights turn off; therefore, ZT23 refers to the time just before the lights go on or just before the onset of the rest phase, and ZT11 refers to the time just before the lights go off or before the onset of the active phase. As in our prior studies, C57BL/6J adult mice were infected at either ZT23 or ZT11 with IAV (H1N1; PR8- 30 PFU) and maintained in 12-hour light-dark (LD) cycling conditions. A subset of mice infected at ZT23 were moved to constant light conditions on day 4 postinfection (p.i.), remaining there throughout the study [referred to as ZT23(LL) mice] (Figure 1A). Light intensity for the LD and LL groups was between 270 and 300 lux. Animals were weighed and monitored daily for 14 days. Our choice of constant light exposure to start on day 4 was driven by our previous work, where we noted the weight trajectory of the ZT11 group started diverging from that of the ZT23 group by day 4, with other signs of inflammation being apparent by day 2 (5). Henceforth, we referred to ZT23(LD) and ZT11(LD) as ZT23 and ZT11, respectively. Additional qualifiers will be used to define how the groups differed from the 12-hour LD cycling.

Consistent with findings from our previous work, mice kept in 12-hour LD cycling and infected at ZT23 had significantly better survival (88.88% in ZT23 vs. 44.82% in ZT11; *P* < 0.01 by Mantel-Cox log-rank test) than the mice infected at ZT11 (Figure 1B). However, the ZT23(LL) group had significantly lower survival [58.33% in ZT23(LL) vs. 88.88% in ZT23; *P* < 0.05 by Mantel-Cox log-rank test] than the ZT23 group (Figure 1B). This was comparable to the mice infected at ZT11 [44.82% survival in ZT11 and 58.33% in ZT23(LL)]. This effect was also reflected in the weight loss trajectory, where the ZT23-infected groups are identical until day 4 p.i. (Figure 1C). However, thereafter, the weight loss was more pronounced in the ZT23(LL) than the ZT23 group, eventually becoming indistinguishable from that of the ZT11 group (Figure 1C). We also monitored these mice for sickness behavior using a previously validated scoring system (Supplemental Table 1; supplemental material available online with this article; https://doi.org/10.1172/JCI193133DS1) based on activity level, behavior, and respiratory distress (5, 6) — the higher the score, the sicker the animal. Like the ZT11 mice, the ZT23(LL) group had consistently higher scores than the ZT23 group, suggesting higher morbidity with constant light exposure (Figure 1D). Upon stratification by sex, we saw similar patterns in both males and females, which is consistent with our previous work (5, 7) (Supplemental Figure 1). Together, these data suggest that disruption of light-dark cycling, even after the initial few days of influenza infection, abrogates the clock-driven time-of-day-specific protection from influenza-induced mortality and morbidity.

### Constant light exposure does not affect ultimate clearance of virus.

**Figure 2:**
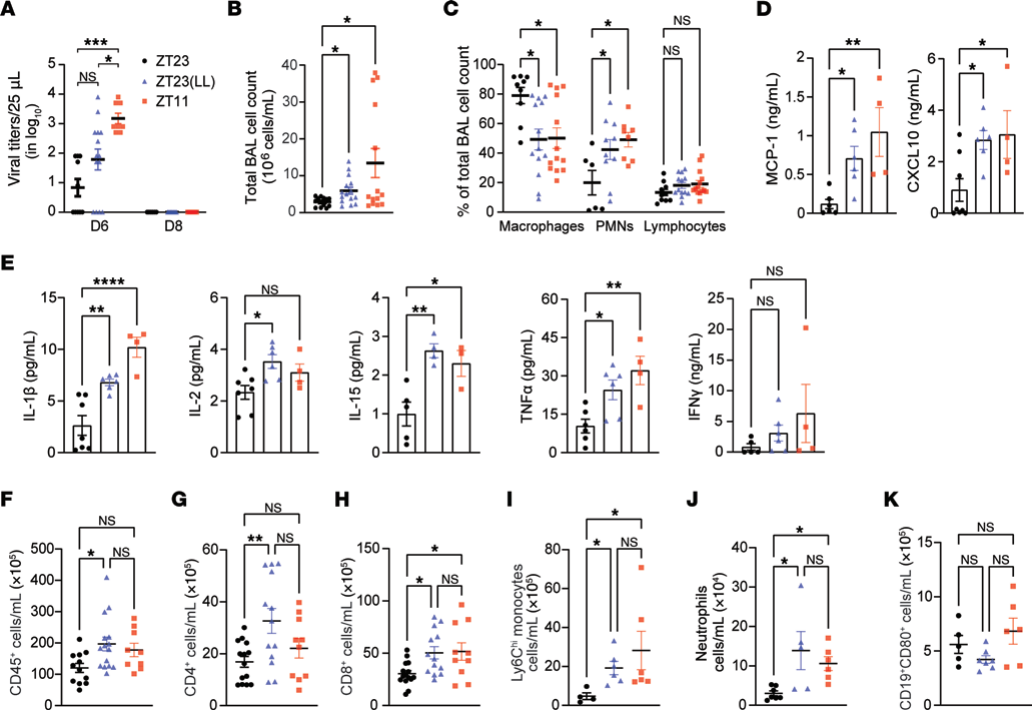
Exposure to constant light following IAV infection caused exaggerated inflammation independent of viral burden. (**A**) Quantification of viral titers (day 6, *n* = 8–14 per group; day 8, *n* = 6–10 per group; *P < 0.05, 1-way ANOVA; ***P < 0.0005, 2-way ANOVA with main effects only). (**B**) Total bronchoalveolar lavage (BAL) on 6 days p.i. (*n* = 13–15 per group; *P < 0.05, 1-way ANOVA, Kruskal Wallis test). (**C**) Differential of the total BAL cells (*n* = 6–13 per group; *P < 0.05, **P < 0.001 2-way ANOVA, mixed effects model). (**D**) Chemokine levels in BAL on day 6 p.i. (*n* = 3–8 per group; *P < 0.05, **P < 0.001, 1-way ANOVA). (**E**) Cytokine levels in BAL on day 6 p.i. (*n* = 3–7 per group; *P < 0.05, **P < 0.001, ****P < 0.0001, 1-way ANOVA). (**F**) Absolute number of CD45^+^ cells. (**G**) Absolute number of CD4^+^ T lymphocytes. (**H**) Absolute number of CD8^+^ T lymphocytes. (**F**–**H**) *n* = 9–15 per group; *P < 0.05, **P < 0.001, 1-way ANOVA. (**I**) Absolute number of Ly6C^hi^ inflammatory monocytes and (**J**) absolute number of Neutrophils. (**K**) Absolute number of B cells (*n* = 4–7 per group *P < 0.05, 1-way ANOVA; Kruskal-Wallis test). All data were pooled from 3–5 independent experiments.

We quantified viral titers in lung tissue to determine whether environmental light disruption affects IAV clearance. Since the disruption of light cycling was introduced on day 4 p.i., viral titers were measured on days 6 and 8 p.i. to allow the host to experience the effects of this perturbation. For this dose of the IAV, we expected days 6 and 8 p.i. to represent time points that follow peak viral titers and likely represent viral clearance. No significant difference in viral burden was observed between the ZT23 and ZT23(LL) groups on either day (Figure 2A). However, the ZT23 group exhibited significantly lower viral titers than the ZT11 group on day 6, consistent with our previous work, adjusted for the initial dose of the virus at infection (5). The ZT23(LL) group had viral titers that fell between the ZT23 and ZT11 groups. However, by day 8, all groups had cleared the virus. These findings suggest that light-dark cycle disruption does not significantly affect IAV clearance.

### Constant light exposure worsens immune-mediated pathology in IAV infection.

Given that the viral titers were comparable in the ZT23(LL) and ZT23 groups despite significantly higher mortality in the former, we next investigated if exposure to constant light worsened lung inflammation. Consistent with our previous work (5), the total bronchoalveolar lavage (BAL) cell count was higher in the mice infected at ZT11 than in those infected at ZT23 (Figure 2B). Interestingly, the ZT23(LL) group mice had higher total BAL cell counts than the ZT23 group, comparable to the ZT11 group (Figure 2B). The higher BAL count in the ZT23(LL) and ZT11 mice was accompanied by a significantly lower percentage of macrophages compared with the ZT23(LD) group (Figure 2C). Next, to test if a short period of LD cycling-driven circadian disruption can result in a state of heightened inflammation in the lungs, we examined BAL from naive mice exposed to constant light for 4 days. We found no differences in the total BAL cell counts between mice maintained in 12-hour LD cycling and those exposed to constant light (Supplemental Figure 2, A and B). Considered together, this supports the idea that the effect of short periods of light disruption is exacerbated in an infected host. The ZT23(LL) and ZT11 groups had significantly more polymorphonuclear cells in the BAL than the ZT23 group (Figure 2C). Furthermore, compared with the ZT23 group, on day 6 p.i., the levels of chemokines, MCP-1 and CXCL10, were significantly higher in the BAL of the ZT23(LL) and ZT11 groups (Figure 2D). The levels of cytokines IL-1β and IL-15 were also significantly higher in both the ZT23(LL) and ZT11 groups than in the ZT23 group, whereas the level of IL-2 was only significantly higher in the ZT23(LL) group (Figure 2E). Interestingly, there was no significant difference in IFN-γ levels across the 3 groups on day 6 p.i. (Figure 2E). Overall, the chemokine/cytokine profile of the ZT23(LL) group was aligned with that of the ZT11 group. Based on these analyses, we hypothesized that the ZT23(LL) group would have more proinflammatory immune populations than the ZT23 group. Therefore, we harvested lungs on day 8 p.i. and compared the immune populations in the 3 groups. Consistent with results from our previous work and our BAL analyses, we found that the ZT23(LL) mice had more leukocytes in the lungs than the ZT23 mice (Figure 2F). Because the ZT23(LL) group had higher levels of MCP-1 and CXCL10 — chemokines that promote monocyte and lymphocyte trafficking, respectively, into inflamed lungs — we enumerated these populations in the lungs (Figure 2, G–K). Ly6C^hi^ inflammatory monocytes (CD45^+^Ly6G^–^CD11b^+^Ly6C^hi^) and T cells (CD45^+^CD4^+^ and CD45^+^CD8^+^) were higher in the ZT23(LL) group than in the ZT23 group (Figure 2, G–I). We posit that the intraparenchymal location and the migration patterns of T cells following influenza A infection explain why the percentage of lymphocytes in the BAL on after infection day 6 was not different for the ZT23 group versus the ZT11 or ZT23(LL) groups in Figure 2B. On the other hand, flow cytometry of lung cells on day 8 revealed an elevation of T cells (Figure 2, G and H). The absolute number of neutrophils (CD45^+^Ly6G^+^CD11b^+^) was also higher in the ZT23(LL) group than in the ZT23 group; however, the number of B cells was comparable across the 3 groups (Figure 2K). Thus, the disruption of environmental lighting leads to an exaggerated inflammatory milieu, marked by high levels of chemokines and accumulation of inflammatory cell populations.

### Constant light exposure worsens lung injury from IAV.

**Figure 3:**
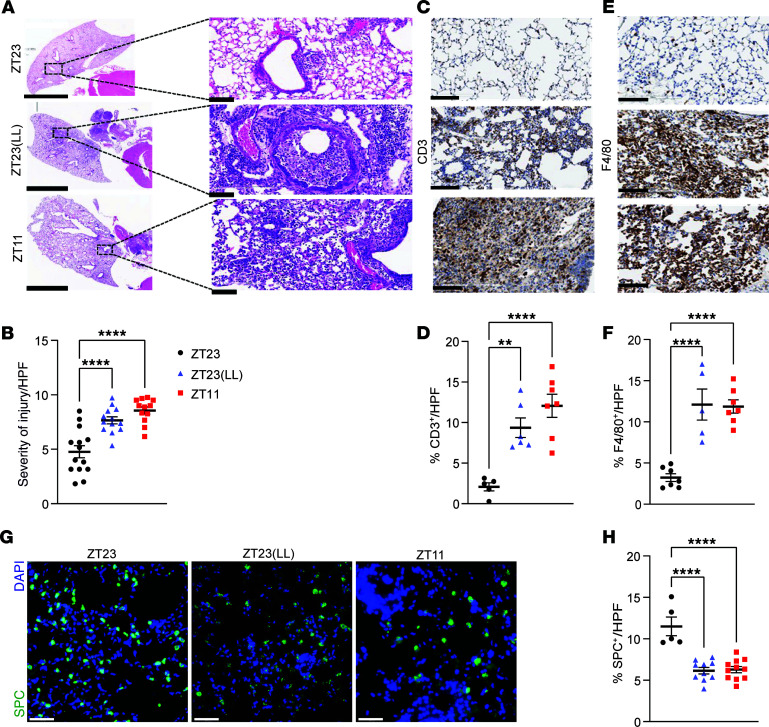
Immunopathology from IAV is worsened by environmental light cycling disruption. (**A**) Representative micrographs of H&E-stained lung sections on day 8 p.i. Left: Scale bar: 5 mm (left); 200 μm (right). Original magnification, ×20 (right). (**B**) Quantification of acute lung injury (*n* = 12–14 per group; ****P < 0.0001, 1-way ANOVA). (**C**) Representative micrographs of CD3-stained lung sections on day 8 p.i. Original magnification, ×20; scale bar: 200 μm. (**D**) Quantification of CD3^+^ cells per HPF (*n* = 5–7 per group; **P < 0.001, ****P < 0.0001, 1-way ANOVA). (**E**) Representative micrographs of F4/80-stained lung sections on day 8 p.i. Original magnification, ×20; Scale bar: 200 μm. (**F**) Quantification of F4/80^+^ cells per HPF (*n* = 5–7 per group; ****P < 0.0001, 1-way ANOVA). (**G**) Representative images of pro-SPC-stained lung sections on day 8 p.i. Original magnification, ×40. (**H**) Quantification of SFTPC^+^ (SPC^+^) alveolar type 2 cells per HPF (*n* = 5–11 per group; ****P < 0.0001 1-way ANOVA). Scale bar: 100 μm. All data were pooled from 5 independent experiments.

To validate the above results, we undertook a histological assessment of lungs harvested from the 3 groups. Consistent with our previous results, using a validated scoring system (5, 6), the ZT23 mice sustained significantly less injury than mice in the ZT11 group (Figure 3, A and B, and Supplemental Table 2). Lung pathology was markedly worse for the ZT23(LL) than the ZT23 group, with higher peribronchial infiltrates, perivascular infiltrates, inflammatory alveolar exudates, and epithelial necrosis (Figure 3, A and B). Furthermore, we validated the results of our immunophenotyping on histology from day 8 p.i. by staining for T lymphocytes (CD3^+^) and macrophages and monocytes (F4/80^+^). The lungs harvested from the ZT23(LL) and ZT11 groups had a significantly higher proportion of CD3^+^ and F4/80^+^ cells per high-power field (HPF) than those of the ZT23 group (Figure 3, C–F, respectively). Next, we investigated if constant light exposure would affect lung regeneration, even beyond the acute immunopathology described above. After severe lung injury, alveolar type 2 (AT2) cells promote the regeneration of the airspace or alveoli (8–11). We found that there were significantly fewer AT2 cells, denoted by SFTPC^+^ cells/HPF, in the lungs of the ZT23(LL) and ZT11 groups than in the ZT23 group (Figure 3, G and H). This could have affected how the lungs regenerate after the initial inflammatory response. Interestingly, we previously showed that the circadian clock, especially the AT2 clock, affects mortality (6), lung regeneration, and repair following IAV infection (7). Although the primary driver for the poorer outcomes in the ZT23(LL) group, like those for the ZT11 group historically, is immune-mediated pathology, a loss of regenerative capacity would likely worsen recovery from IAV, leading to long-term morbidity.

### Light disruption is effective during a specific window of vulnerability following influenza A infection.

**Figure 4:**
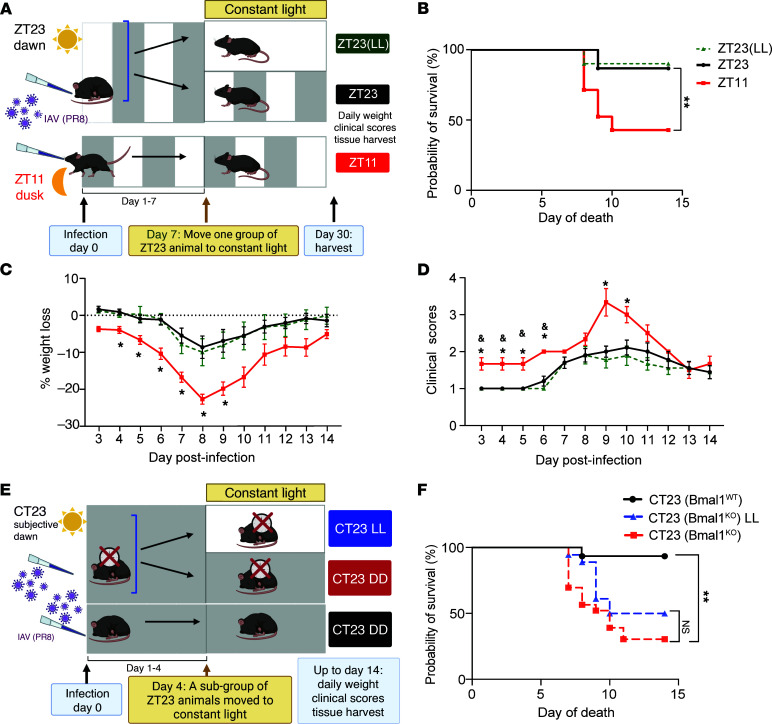
A specific window of vulnerability to light disruption following influenza A infection existed. (**A**) Experimental model. (**B**) Survival (*n* = 10–21 per group, **P < 0.001 log-rank test). (**C**) Weight loss trajectory (*n* = 10–21 per group, *P < 0.05 ANOVA for repeated measures) following IAV infection. (**D**) Average clinical score (*n* = 10–21 per group, *P < 0.05 ANOVA for repeated measures). ZT23 vs. ZT11 comparisons are indicated with an *; ZT23(LL) day 7 vs. ZT11 comparisons are indicated with a &. (**E**) Experimental model. (**F**) Survival (*n* = 10–18 per group, **P < 0.001 log-rank test). All data were pooled from 3 independent experiments.

Having established the need for synchronizing the influence of entraining photic cues in maintaining the clock-driven protection from IAV in the early phase of influenza, we asked if there exists a window of vulnerability to light disruption following influenza infection. At the range of the doses used for the infection, we expected the peak viral titers to be around day 4 and clearance coinciding with day 7–8. Therefore, we infected mice at either ZT23 or ZT11 and maintained them in 12-hour LD cycling conditions until day 7 p.i. At this time, a subset of the ZT23 groups was moved to constant light, referred to as the ZT23(LL-DAY7) group, instead of day 4, as in earlier experiments (Figure 4A).

Mice from the ZT23 group had significantly lower mortality than mice infected at ZT11. However, unlike the results from constant light exposure on day 4 p.i., the time-of-day difference in outcomes was not lost when animals were moved to constant light on day 7 p.i. [Figure 4B; 90% survival in ZT23 versus 86.66% survival in ZT23(LL-DAY7) and 42.8% in ZT11]. Furthermore, the weight loss trajectory and morbidity of the ZT23(LL-DAY7) group were comparable to that of the ZT23 group (Figure 4, C and D), suggesting that there was a window of vulnerability to LD disruption following influenza infection.

### The central clock mediates the abrogation of time-of-day-specific protection.

To investigate if the central clock mediates the loss of time-of-day-specific protection in influenza infection in mice exposed to constant light, we analyzed the locomotor activity as a read out for the former. Since singly housing mice (necessary for locomotor activity monitoring) after IAV infection could worsen outcomes, we used naive mice in this experiment. Mice were exposed to 4 weeks of LD cycling and then moved to constant light conditions for 3 weeks. We found that the mice in LD cycling showed clear demarcation between active and rest phases, coinciding with the dark and light phases of the 24-hour cycle (Supplemental Figure 3). The mice exposed to constant light displayed erratic onsets of activity, eventually becoming completely arrhythmic after being exposed to constant light for 3 weeks (Supplemental Figure 3A). Mice kept in continuous light, although arrhythmic, had comparable activity levels, supporting the idea that constant light led to a loss of rhythmicity but did not impair overall activity level (Supplemental Figure 3, B and C). Prolonged constant light exposure in an uninfected host is also associated with increased stress response (12, 13). This response may include neurohumoral alteration (14–16), immune perturbations (17–19), metabolic dysregulation (20, 21), and adverse behavioral changes (15, 22). Many of these effects are often attributed to circadian disruption. To test whether the increased mortality observed under constant light is due to circadian-independent stress mechanisms, we repeated this experiment in clock mutant mice. Given our context-specific and short duration of LL exposure, we hypothesized that the effect of LL in our influenza model mediates its effect mainly through circadian disruption, rather than additional stress independent of circadian disruption.

To test this, we used *iBmal1^–/–^* (*Bmal1^fl/fl^ ERT2Cre^+^*) mice, in which the clock has been genetically disrupted globally. These mice are arrhythmic in constant darkness; henceforth, they are abbreviated as DD (23). Previously, we have shown that these *iBmal1^–/–^* mice do not have clock-driven time-of-day-specific protection against IAV (5). Initially, we tested the effect of LD cycling on WT and *iBmal1^–/–^* mice to maintain consistency with our experimental design, as outlined in Figure 1. However, we found that *Bmal1^–/–^* mice under LD conditions showed a reversal of the time of day-specific protection compared with WT mice, with *iBmal1^–/–^* mice infected at ZT11 having better outcomes than *iBmal1^–/–^* mice infected at ZT23 (Supplemental Figure 4). This would suggest that, in the absence of the molecular clock, LD cycling primes processes allowing a reversal but not an abrogation of the time-of-day protection. Thus, based on our previously published work, we tested the *Bmal1^–/–^* mice in DD conditions (5). In circadian terminology, CT time refers to the same times as ZT but under DD conditions. Therefore, CT23 represents subjective dawn. We infected *Bmal1^–/–^* mice and their cre^neg^ littermates maintained in DD conditions. On day 4 p.i., a subset of *Bmal1^–/–^* mice were moved from constant darkness to constant light conditions (Figure 4E). We found that, consistent with our previous results (5), *Bmal1^–/–^* mice infected at CT23 experienced significantly higher mortality than wild-type littermates (*P* < 0.001, Mantel-Cox log-rank test; Figure 4F). However, while *Bmal1^–/–^* mice exposed to constant light still had higher mortality than the WT littermates, there was no difference in the outcomes of *Bmal1^–/–^* mice in the 2 lighting conditions [survival of 31% in *iBmal1^–/–^* CT23 and 50% in *iBmal1^–/–^* CT23(LL-D4)], suggesting that constant light did not worsen the outcomes over that attributed to loss of the clock.

### Global immune activation observed in the dawn-infected group when exposed to constant light after infection.

**Figure 5:**
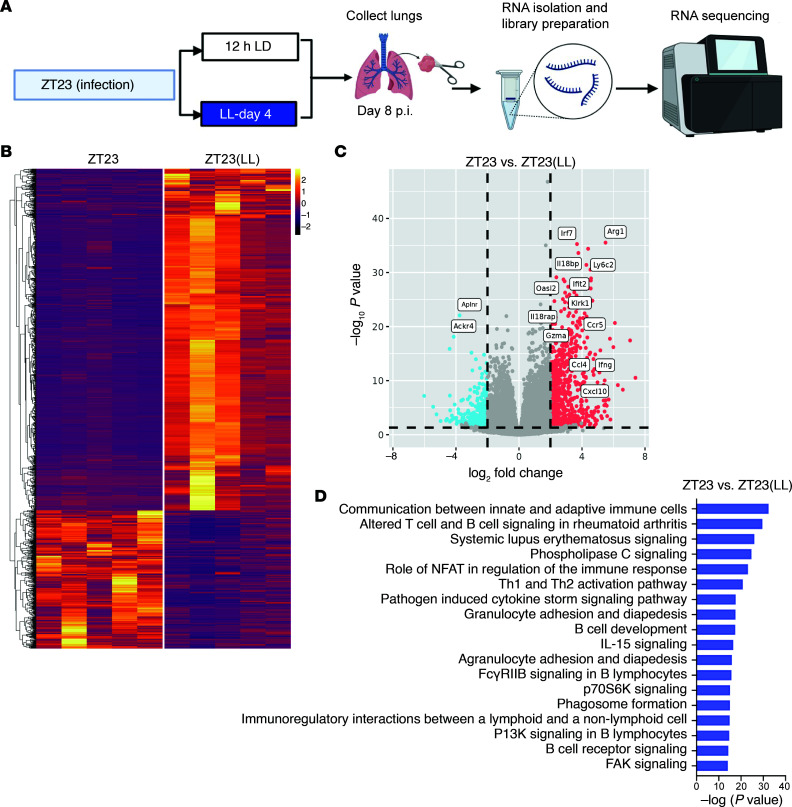
Transcriptomic analyses showed global immune activation in the ZT23 and ZT23(LL) groups. (**A**) Schematic of bulk RNA-Seq processing on day 8 p.i. lungs. (**B**) Heatmaps of all differentially regulated genes. (**C**) Volcano plots showing upregulated and downregulated genes. In each volcano plot, the horizontal dotted line represents a *P*_adj_ = 0.05, and the vertical dotted line represents a log (fold change) >2 or <–2. (**D**) Plot of log-adjusted fold change for ZT23 vs. ZT23(LL) shows the directionality of the most differentially expressed genes. *n* = 5 per group, all females. All data were pooled from 3 independent experiments.

To determine what pathways resulted in worse outcomes in the ZT23(LL) group, we performed transcriptional profiling on lungs harvested on day 8 p.i from both ZT23 and ZT23(LL) (*n* = 5/ group; all females) (Figure 5A). Environmental light disruption following influenza infection resulted in disparate transcriptional phenotypes (Figure 5B). 1,125 genes were differentially expressed between the 2 groups, with 801 genes upregulated and 324 genes downregulated in the ZT23(LL) group relative to the ZT23 group. Several genes associated with the migration of leukocytes (*Ccr5*, *Ccl4*, *Cxcl10*) into the lung and exaggerated immune activation (*Il18rap*, *Gzma*, *Ly6c2*), were upregulated in the ZT23(LL) group compared with the ZT23 group (Figure 5C). Further pathway analysis revealed that while those involved in innate activation (granulocyte diapedesis, IL-15 signaling, cytokine storm) were upregulated in the ZT23(LL) group relative to the ZT23 group, the most highly significant pathways involved innate to adaptive communication (communication between innate and adaptive cells) and activation of the adaptive immune system (B cell activation as in systemic lupus, altered T and B cells signaling, NFAT signaling) (Figure 5D). Overall, these pathways supported our findings of excessive immune cells, especially lymphocytes and monocytes, in the lungs of the ZT23(LL) group compared with the ZT23 group. Thus, even a short duration of light-based circadian disruption caused profound alteration of the immune response.

### Transcriptomic parallels between the effect of light disruption and time-of-day effects.

**Figure 6:**
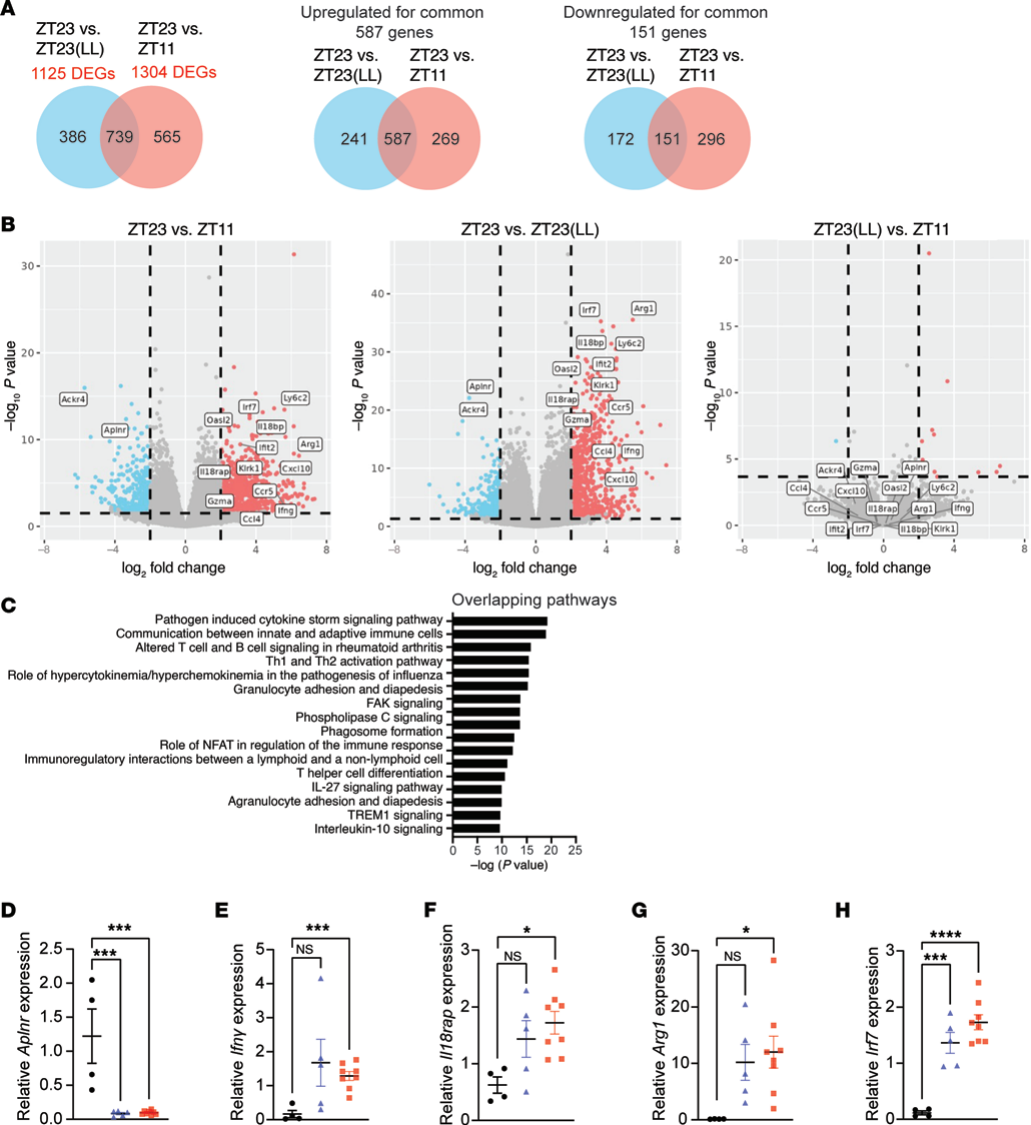
Transcriptomic analyses showed global immune activation in the ZT23(LL) and ZT11 LD groups. (**A**) Venn diagram (sizes not to scale) depicting the number of differentially expressed genes. (**B**) Volcano plots showing upregulated and downregulated genes. In each volcano plot, the horizontal dotted line represents a *P*_adj_ = 0.05, and the vertical dotted line represents a log (fold change) >2 or <–2. (**C**) Plot of log-adjusted fold change for overlapping pathways between ZT23 LD vs. ZT23(LL) and ZT23 LD vs. ZT11 LD. (**D**–**H**) Relative gene expression of selected genes as a confirmation for bulk RNA-Seq. *n* = 4–8 per group, *P < 0.05, **P < 0.001, ***P < 0.0005, ****P < 0.0001; 1-way ANOVA. All data were pooled from 3 independent experiments.

While we have shown that the ZT11 and ZT23(LL) groups both show excessive immune infiltration and subsequent tissue destruction (Figures 2 and 3), we wanted to test if the mechanism(s) underlying the poorer outcomes in both these groups were indeed similar. For this, we expanded our transcriptomic study to include 3 groups — ZT23, ZT11, and ZT23(LL) (Figure 6A). There were 1,304 differentially expressed (DE) genes when comparing the ZT23 group with the ZT11 group. Of these, 856 genes were upregulated and 448 genes were downregulated in the ZT11 group relative to the ZT23 group. Of the 1,304 DE genes, 739 genes were common to the comparison between ZT23 vs. ZT23(LL) (Figure 6A). Of these 739 shared DE genes, 587 genes were upregulated, while 151 genes were downregulated in both ZT23(LL) and ZT11 groups relative to the ZT23 group. Only one low-expression gene (*Oxtr*) was downregulated in the ZT23(LL) group and upregulated in the ZT11 group. Thus, not only was there significant overlap in the DE genes (Figure 6B), but their directionality was also conserved across the 2 comparisons — that of ZT23(LL) versus ZT23 and ZT11 versus ZT23, respectively. To understand how the cellular composition of the lungs from each of these 3 groups affected our transcriptomic profile, we used another dataset from day 8 p.i., where single-nuclear RNA-Seq (sn-RNA-Seq) analyses had been performed. This allowed us to use deconvolution algorithms to infer the cellular composition (24). As expected, the ZT23 groups had a markedly lower proportion of immune cells than the ZT23(LL) or ZT11 groups (Supplemental Figure 5, A and B). Next, we compared the ZT23(LL) with the ZT11 group to further confirm the mechanistic overlap. We found only 13 DE genes with a 2-fold change in expression between these 2 groups; of these, many were circadian genes like *Bmal1* (*Arntl*), *Dbp*, and *Npas2*, whose expression oscillates across the day and is expected to be disrupted by constant light (Figure 6C). In further analyses, as expected, various innate immune pathways, namely, “cell adhesion and diapedesis” and “the role of hypercytokinemia/hypercytokinemia in the pathogenesis of influenza,” were enriched and conserved in both comparisons (Figure 6C). Pathways involved in adaptive immune activation were also enriched in the ZT23(LL) and ZT11 groups relative to the ZT23 group (Figure 6C). Genes involved in leukocyte migration (*Ccl4*, *Ccr5*) and activation of the immune system (*Ifng*, *Klrk1*, *Gzma*, *Il18rap*) were all higher in the ZT23(LL) and ZT11 groups compared with the ZT23 group. We confirmed changes in expression in some of the key genes by qPCR (Figure 6, D–H). Together, these results suggested that LD-cycling perturbation following influenza impairs lymphocyte and monocyte diapedesis and results in the clock-regulated less favorable time of day. Next, we blocked the migration of leukocytes into the lungs in the ZT23(LL) groups by administering anti-leukocyte function–associated antigen (LFA) antibody on day 4 p.i., a time coinciding with their transition from LD cycling to constant light (Supplemental Figure 6A). Interestingly, we observed that blocking leukocyte migration did not improve the outcomes in the ZT23(LL) group [Supplemental Figure 6B; 93.3% survival in ZT23 vs. 54.5% in ZT11, vs. 65% in ZT23(LL) vs. 53.8% in ZT23(LL) with anti-LFA]. This raises 2 possibilities that are not mutually exclusive. First, some leukocyte migration may be beneficial to the host, and inhibiting all leukocyte trafficking while protecting from the effects of light disruption might make the host more vulnerable owing to the absence of the protective cell populations, at least when inhibited early on day 4 p.i. The second and the more speculative possibility is that the immune cells are merely responding to the signals emanating from the lung parenchyma in response to the light disruption; therefore, blocking leukocyte migration itself has minimal independent effect on mortality. Regardless, from these experiments, we concluded that the processes affected by light disruption are usually regulated by the clock to confer time-of-day-specific protection.

### Cycling of food availability restores the time-of-day-specific protection from influenza A infection in the face of environmental light disruption.

**Figure 7:**
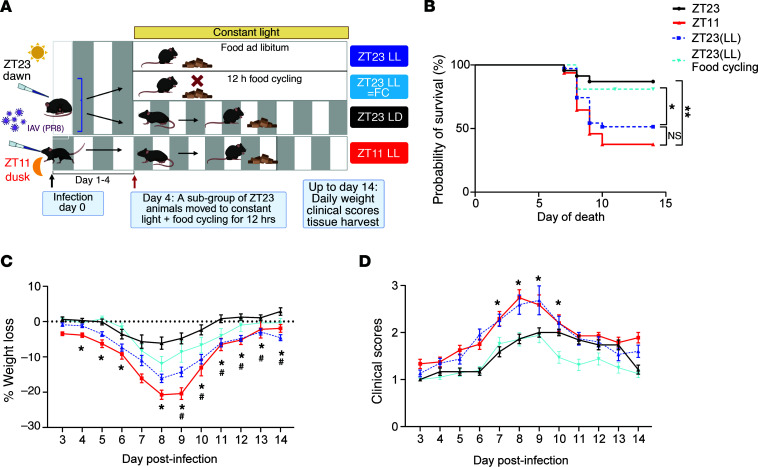
Food cycling rescued the loss of time-of-day protection from influenza A following environmental light disruption. (**A**) Experimental model. (**B**) Survival (*n* = 21–50 per group; **P < 0.01 log-rank test from 3 independent experiments, *P < 0.05 log-rank test from 3 independent experiments). (**C**) Weight loss trajectory (*n* = 21–50 per group, *P < 0.05, ANOVA for repeated measures; ^#^*P* < 0.001, ANOVA for repeated measures) following IAV. (**D**) Average clinical score. (*n* = 21–50 per group, *P < 0.05 ANOVA for repeated measures) following IAV infection. All data were pooled from 3 independent experiments. ZT23 vs. ZT11 comparisons are indicated with an *; ZT23 vs. ZT23(LL-food cycling) comparisons are indicated with a #.

While light is the primary external cue that entrains and synchronizes the central and peripheral clocks, other external cues like exercise and meal timing may significantly affect peripheral clocks (25). To investigate whether the poor outcomes caused by environmental light perturbation could be rescued by using a different entrainment cue, we considered a role for food cycling (FC) in the presence of environmental light perturbation. We followed the experimental design in Figure 1A, where mice were infected at either ZT23 or ZT11 and a subset of mice infected at ZT23 were moved to constant light on day 4 after infection. Within the ZT23(LL) group, a subset of mice was subjected to food cycling, wherein food was provided from ZT12 to ZT0 each day for the ZT23(LL-food cycling) group, while the other had access to food ad libitum [ZT23(LL)]; all groups had unrestricted access to water (Figure 7A). Consistent with our previous results, the ZT23(LL) with food ad libitum group had higher mortality than the ZT23 group. As in previous experiments, the ZT23 group had significantly better survival than the ZT11 group (Figure 7B, 87% survival in ZT23 versus 37.5% survival in ZT11; *P* < 0.001 by Mantel-Cox log-rank test). Likewise, the exposure to constant light significantly reduced the survival in the ZT23(LL) group [Figure 7B, 51% survival in ZT23(LL) vs. 86% survival in the ZT23 group, *P* < 0.05 by Mantel-Cox log-rank test]. Interestingly, the mice from the ZT23(LL-food cycling) group showed rescue of the survival advantage associated with infection at ZT23 that had been lost in the ZT23(LL) group [Figure 7B, survival of 81% in ZT23(LL-food cycling) vs. 51% in ZT23(LL); *P* < 0.05, Mantel-Cox Log-rank test]. The mice in the food-cycling group also lost less weight than those in the ZT23(LL) group (Figure 7C). The ZT23(LL-food cycling) groups also exhibited lower clinical scores, suggesting less morbidity than that in the ZT23(LL) group (Figure 7D). These findings led to the conclusion that, while LD cycling and meal timing are important zeitgebers affecting the host’s circadian rhythms, the latter is hierarchically a more potent entraining factor for optimal host response to influenza infection.

## Discussion

While we and others have demonstrated time-of-day-specific protection conferred by the circadian clock (5, 26, 27), our study uncovers how common zeitgebers interact dynamically after the initial infection to shape the host’s response to the pathogen. Circadian disruption is known to worsen outcomes for many health conditions, including infection; most often, these are genetic or environmental disruptions that predate the infection (27–30). When time-of-day studies are undertaken for infections or other discrete stimuli, the sentinel event is deemed to occur on the day of the infection. Prior studies have not clearly established whether circadian regulatory mechanisms continue to influence host protection following infection. We have previously shown that mice infected at ZT11 had a 3-fold higher mortality rate than mice infected at ZT23 (5). Since the peak mortality from influenza infection is between days 8 and 10 in mice, questions have remained on whether it is the early signaling following infection or an effective but continuing cascade of well-regulated immune response to IAV that drives the time-of-day-specific protection. While the effect of longstanding disruption of LD cycling, as simulated by chronic jet lag, on health outcomes (31) is well known, the impact of short-term disruption of LD cycling after acute infection is poorly understood. Our study shows that this time-of-day-specific protection in the ZT23 group is lost when mice are subjected to circadian disruption via constant light exposure. The effect of circadian disruption, as seen with constant light exposure, has been studied in other contexts (32, 33). However, the role of a short duration of light exposure on acute lung injury has not been investigated before. Some studies, although not investigating the lungs, used >4 weeks of constant light exposure to define outcomes (21, 34) or higher-intensity light cycling at baseline (35). We found that even a short duration of constant light exposure in an infected animal worsened lung inflammation. Given the translational scope of the work, we hoped that starting constant light a few days after the infection, rather than preceding the infection, would better simulate the effect of circadian disruptions on host response, whether in hospital settings or otherwise.

Our locomotor activity analyses documenting the disruptive effect of constant light on circadian rhythms were done in the naive host and with a substantially longer duration of constant light exposure (Figure 4E). We found it technically challenging to house mice singly (36, 37) for sustained periods (that would be necessary for doing locomotor activity analyses), especially given the trajectory of the acute illness of influenza infection. Although this protocol clearly demonstrated loss of rhythmicity, we verified the role of the clock in the constant light paradigm by conducting light disruption in a clock-mutant background. We showed that, in mice with genetically disrupted clocks, constant light exposure did not worsen outcomes following IAV. Our work on the effect of LD cycling on the *Bmal1^fl/fl^ ERT2Cre^+^* mice also highlights the complexity of the entrainment against a background of a lack of endogenous clock in the clock-disrupted mice.

Importantly, we show that food cycling–based entrainment was able to restore the clock-driven advantage lost due to the disruption of photic cues in influenza infection. Because timed feeding does not entrain the SCN clock (38), our findings would support the idea that metabolic cues are more critical for the entrainment of peripheral clocks than SCN-driven signals entrained by photic cues when facing an energy-demanding function like fighting an infection. The responsiveness of tissue-specific clocks to food cycling has been demonstrated for the liver clock (33, 38–40) and is supported by reports from the lung (41, 42); however, these studies typically involve longer entrainment and uninfected hosts. The protective effect of food cycling that we observed could be mediated by food-driven entrainment of peripheral clocks in the liver, lung, or immune system. Future studies dissecting the crosstalk between the central and peripheral clocks based on entraining cues will be critical for translating these findings clinically. However, the fact that a short duration of constant light and food cycling is sufficient to change the trajectory in mice infected at ZT23 underscores the importance of maintaining “entraining cues” in acutely ill hosts. Given the acute immune and metabolic stress from the infection, such a host may be especially vulnerable to circadian perturbation.

We recognize the inherent limitation of translating findings from circadian studies in nocturnal mice to diurnal humans. Nevertheless, direct circadian studies in humans are extremely challenging, making well-controlled animal models essential for establishing biological principles. The current study provides excellent proof-of-concept evidence that disruption of environmental cues compromises host defense against influenza — a finding with clear implications for human health. While we cannot control the time of infection in clinical settings, the time-of-day studies remain a valuable tool for uncovering mechanisms by which circadian health shapes immune responses. Moreover, although our work focused on young adult mice, its relevance may be even greater for populations at the extremes of age (43–45) who are especially vulnerable to both circadian disruption and severe viral infections (46, 47). Indeed, strengthening rhythms in older animals could be beneficial for several reasons, and timed interventions will likely also be effective.

Our findings are of particular relevance for hospitalized patients. Chronic circadian disruption, commonly seen in shift workers, is a known risk factor for cancers, hypertension, diabetes mellitus, and metabolic syndrome (4). However, circadian disruption is pervasive in hospitals and intensive care units (48–50). LD cycling is frequently disrupted in these units, with dim and limited-spectrum light exposure during the day and erratic but more light exposure at night (51). While such disruption has been linked to delirium or other neuropsychiatric symptoms, to our knowledge, we now show deleterious effects on the pulmonary immune response. Furthermore, the effect of visible light on organismal physiology also depends on the spectral composition and is mediated via both circadian and noncircadian pathways (52). For example, blue light affects the metabolism in adipocytes in an Opsin-3–dependent manner, completely independent of circadian pathways (53, 54). Given the challenges of simulating erratic LD cycling, we used constant light as a tractable approach reflecting one extreme of environmental disturbance — and observed adverse immune responses within days of infection. Though erratic light cycling may differ in its effects, both erratic and constant light exposure represent clinically relevant forms of photic cue-mediated circadian disruption. Thus, our data suggest that circadian health may be influenced by well-timed environmental cues and is a modifiable factor with potential to drive outcomes in respiratory viral infection.

When our findings are considered altogether, our study argues for incorporating circadian-sensitive practices on hospital floors and intensive care units. These may include, but are not limited to, light-cycling and meal-timing interventions as strategies to bolster immune response and hasten recovery. Beyond mechanistic insights, this study highlights the critical need to consider circadian health as a core component of supportive care in critical illness, opening avenues for simple, noninvasive interventions with the potential to improve patient outcomes.

## Methods

### Sex as a biological variable.

Our study examined both male and female mice, and similar findings were reported in both sexes (Supplemental Figure 1). All experiments were conducted with age- (10–20 weeks) and sex-matched mice. Male and female mice were used in approximately equal proportions for individual experiments unless specified. Given that there were no differences in the overall phenotype with constant light exposure, only female mice were used for the RNA-Seq experiment to minimize any variability.

### Mice, virus, infection, constant light exposure, and food cycling.

Specific pathogen–free 8- to 16-week-old C57BL/6J mice (both males and females) were placed in circadian cabinets on reverse light-dark cycles as described previously (5). This allowed us to simultaneously infect mice or harvest tissues across all experimental groups. After 4–6 weeks of acclimatization in these cabinets, mice were anesthetized lightly with isoflurane prior to being infected intranasally with mouse-adapted influenza virus (PR8). Given the use of circadian cabinets, mice were simultaneously infected at either ZT23 or ZT11 and maintained in 12-hour light-dark cycle. The dose chosen was likely to cause >50% mortality when mice were infected at ZT11. Where relevant, on days 4 or 7 p.i., a subset of ZT23-LD mice was moved to constant light until the end of the study. Animals were weighed and scored based on a previously validated scoring criterion each day. For a subset of the ZT23 LL mice, the food was cycled to a 12-hour period, coinciding with their active and rest phases, which had them remain in 12-hour LD conditions. Food trays were manually removed for the food cycling experiments. The food trays in non-food-cycled study groups were handled but not removed to control for any stress associated with this procedure. C57BL/6J mice were either purchased from The Jackson Laboratory or bred in-house.

### Genetic mouse mutants.

Inducible Bmal1^–/–^ mice were generated using 2-month-old *Bmal1^fl/fl^ ERT2Cre^+^* mice treated with 5 mg (in 50 μL) tamoxifen via oral gavage daily for 5 consecutive days (5). *Bmal1^fl/fl^ Cre^neg^* littermates treated with tamoxifen were used as controls. They were exposed to constant darkness for 1–2 days before infection with IAV at CT23. Both males and females were used in equal proportions in the mentioned experiment. These mice were originally purchased from The Jackson Laboratory and then bred up for experiments.

### Viral titration.

Lungs were harvested and homogenized in 0.1% PBS-gelatin. Influenza virus was detected by using MDCK cells (gift from Scott Hensley’s group at the University of Pennsylvania; originally purchased from ATCC, PTA-6500). MDCK cells were overlaid with 1:10 dilutions of the homogenized lungs and incubated at 37°C; 5% CO_2_ for 1 hour to allow the virus to infect the MDCK cells. Thereafter, 175 μL of tissue culture medium supplemented with 2 μg/mL of TPCK-treated trypsin was added, and cells were incubated for another 72 hours at 37°C; 5% CO_2_ before viral titration. After 72 hours, hemagglutination of turkey red blood cells (RBCs) was tested by collecting 50 μL of the medium from the infected MDCK plate to detect and quantify the virus. The hemagglutination of RBCs confirmed the presence of the virus particles (5).

### Lung histology, immunofluorescence, and BAL cytology.

The trachea of mice was cannulated, and lungs were gently lavaged. The supernatant from the first pass was collected and flash-frozen for ELISA. The cells from all 4 passes were pooled and counted using a Nexcelcom cell counter. Cytospins of BAL cells were stained with Hemacolor (Sigma-Aldrich, 65044-93). The lungs were perfused with PBS, followed by inflation with 10% buffered formalin and then fixing in 10% buffered formalin for 24–48 hours. The pathology core at Children’s Hospital of Philadelphia paraffin-embedded and stained the lungs for H&E stain. Using a previously validated scoring method (5), scoring was performed with scorers remaining blinded to the group assignment. Briefly, 5 lung fields selected at random at ×20 magnification were scored based on a scale of 0–3 for the following: (a) peribronchial infiltrates, (b) perivascular infiltrates, (c) alveolar exudates, and (d) epithelial damage of medium-sized airways.

Paraffin-embedded lung sections were stained for macrophages and monocytes (F4/80) and lymphocyte (CD3) populations using immunohistochemistry (IHC) by the CHOP pathology core. The primary antibody used for macrophage staining was F4/80 (D2S9R) XP Rabbit mAB (Cell Signaling; 70076) and Recombinant anti-CD3 epsilon antibody (EPR20752) (abcam; ab215212) was used for staining the lymphoid population. All stained slides were digitally scanned at ×20 using an Aperio CS-O slide scanner (Leica Biosystems). The lungs were annotated and analyzed using the Nuclear v9 macros from Aperio Imagescope software for F4/80 and CD3 analysis. Quantitation was performed using the Aperio Imagescope software. Data represent percentage positive nuclei (for F4/80 and CD3^+^) per HPF.

For immunofluorescence staining, the paraffin-embedded lung sections were stained with primary antibody SFTPC (1:100, anti-pro surfactant protein, rabbit, AB3786 Millipore) at 4°C overnight, followed by secondary antibody donkey anti-rabbit IgG (H+L) Alexa Fluor 488 (A21206; Invitrogen Antibodies, Thermo Fisher) at 1:250 dilution. The slides were incubated with DAPI (0.2 μg/mL) (Thermo Fisher; 1738176) for 10 minutes. VectaMount AQ (Vector Laboratories Inc.; H-5501) was used to mount the slides. The histological and cytological scoring of all the samples was performed in a blinded fashion. To quantify SFTPC^+^ cells after influenza, images were captured at ×20 on a Leica DM6 fluorescent microscope, and then cells were counted from >4 random sections per sample. The Cell Counter plug-in on ImageJ (NIH) was used to count SFTPC^+^ cells. In total, >1,000 cells were counted from *n* = 3–5 sections per group.

### ELISA.

BAL from the first pass as described above on day 6 p.i. or lung homogenate in 0.1% gelatin on day 8 p.i. was sent for ELISA analysis to the Human Immunology Core at the University of Pennsylvania for 9-plex cytokine array analysis. MCP-1, IP-10, IL-2, IL-1β, IL-15, and TNF-α were measured using a custom ELISA kit (Milliplex Mouse Cytokine/Chemokine Magnetic Bead Panel; MCYTOMAG-70K-09C; Millipore SIGMA). Chemokines and cytokines were measured in 25 μL of BAL per sample in duplicates, and the protocol for 9-plex Luminex Performance Assay was performed according to the manufacturer’s protocol (Millipore SIGMA). FLEXMAP 3D (Bio-Rad) was used to acquire data. Luminex x Potent 4.2 and Bio-Plex Manager 6.1 software (Bio-Rad) were used to analyze data.

### Locomotor activity.

Wireless infrared motion sensors (Actimetrics) were used to measure locomotor activity of singly housed mice before IAV infection. Once the uninfected mice were acclimatized to the circadian cabinets, their activity was measured in 12-hour light-dark cycles for 4 weeks. After 4 weeks, the mice were exposed to constant light for another 3 weeks, and actogram data were collected. Parameters of the animals’ rhythms, like amplitude, period, phase, average activity count, and percentage of variance, were analyzed using Clocklab software.

### RNA extraction and bulk RNA-Seq.

The bulk transcriptomic analyses were performed on female mice only, since sex-stratification confirmed that male and female mice exhibited equivalent circadian patterns in inflammatory (5) and repair-associated phenotypes (7). Similar results were also seen in the current model (Supplemental Figure 1). Restricting to a single sex reduced biological variability while preserving interpretability. TRIzol (Life Technologies) was used to extract and isolate RNA from the inferior lobe of the mouse lung. The RNeasy Mini Elute Clean Up Kit (Qiagen) was used to purify the RNA further as per the manufacturer’s protocol. The NanoDrop ND-1000 spectrophotometer (NanoDrop Technologies Inc.) was used to assess the quality and quantity of the extracted RNA. Only samples with RINS >7 after QC were sent for sequencing to Azenta Life Sciences. Sequencing was done using an Illumina NovaSeq X with Poly A selection to generate 2x 150 strand-specific paired-end reads. Twenty million paired-end reads were obtained per sample. A mouse reference genome on an in-house resampling-based normalization and quantification pipeline aligned the samples. They were further compared with existing gene annotations (ENSEMBL), and loci and isoforms were identified. A false discovery rate–based control for multiple testing was used to identify differentially expressed genes. Finally, to assess fully the effects on key pathways and mediators, Ingenuity and GSEA were used.

### sn-RNA-Seq.

Lungs were harvested on day 8 p.i. Nuclear extraction and next-generation sequencing libraries were prepared using the 10x Genomics single cell 3′ v4 (polyA) reagent kit following the manufacturer’s protocol at Azenta Life Sciences. Libraries were uniquely indexed, pooled, and sequenced on an Illumina sequencer. Libraries were sequenced in a paired-end, dual-index sequencing run targeting 20,000 mean reads per cell. Demultiplexing and mapping of reads to the mouse mm10 genome to generate feature barcode matrices was performed using the Cell Ranger pipeline (v9.0.0, 10x Genomics) with the “include introns” option. Feature barcode count matrices were corrected for ambient RNA contamination using SoupX (v1.6.2), and adjusted expression matrices were imported into Seurat (v5.0.3) within the R statistical computing environment (v4.4.0). Nuclei expressing less than 7,000 features, between 800 and 40,000 unique molecular indexes, and less than 10% mitochondrial content were retained. Potential doublets were detected and removed using scDblFinder (v1.18.0). The filtered dataset was log normalized, scaled while regressing out mitochondrial content, and the top 2,000 highly variable features were utilized as input for PCA. The top 20 PCs were used downstream in uniform manifold approximation and projection (UMAP) and graph-based clustering using the “FindClusters” function within Seurat and a resolution of 0.8. Subsequent clusters and cell types were annotated using a publicly available lung single-cell RNA-Seq dataset by Niethamer et al. (55) as a reference within the singleR package (v2.6.0) and compared with additional canonical marker genes.

### Deconvolution procedures.

To determine the cell-type abundances in the bulk RNA-Seq datasets, we employed a reference sn-RNA-Seq dataset (available in the Gene Expression Omnibus under accession GSE296722) of mouse lung at day 8 after influenza infection. This reference dataset had been processed using the cellranger v9.0.0 pipeline using the mm10 genome, and cell types were annotated using a published mouse lung dataset, also following influenza infection with the same viral strain. The MuSiC method was then used to deconvolve the bulk RNA-Seq datasets by cell type (24). Because the MuSiC method uses replicate reference datasets, we randomly subsampled each cell type into 2 “replicate” data types before running MuSiC. After deconvolution, cell types were grouped into endothelial, epithelial, mesenchymal, or immune cells.

### Flow cytometry.

As described previously, the lungs were digested using DNAse II (Roche) and Liberase (II) at 37°C for 30 minutes after perfusing the lungs with PBS through the right ventricle. The dissociated tissue was then passed through a 70 μm cell strainer, followed by centrifugation and RBC lysis. After washing and resuspending the cells in PBS, 3 × 10^6^ cells were blocked with anti-CD16/32 antibody and first stained with Live/Dead fixability dyes for 30 minutes. Following washing, the cells were stained with desired antibodies on ice for 30 minutes. Cells were fixed in 2% paraformaldehyde. Flow cytometric data were obtained using an LSR Fortessa cytometer and analyzed using FlowJo software (Tree Star Inc.). All cells were pregated on size as singlets, live cells. All subsequent gating was done on CD45^+^ cells only.

### Statement on rigor and reproducibility.

Mice were either purchased from The Jackson Laboratory or bred in-house. The reported findings summarize the results from 3–6 independent experiments.

### Statistics.

GraphPad (Prism V9 and V10) was used for all statistical analyses. In experiments with more than 2 groups and normally distributed data, a 1-way or 2-way ANOVA was performed. Šídák’s correction was used for multiple comparisons. When data were not normally distributed, we used Mann-Whitney or Kruskal-Wallis tests. Most data are represented as mean ± SEM, except for data obtained from objective histological scoring, which was presented as median ± interquartile range. *P* values of less than 0.05 were considered significant.

### Study approval.

Approval from the Children’s Hospital of Philadelphia Institutional Animal Care and Use Committee was obtained for all animal studies, and the stipulations were met according to the *Guide for the Care and Use of Laboratory Animals* (National Academies Press, 2011).

### Data availability.

The sn-RNA-Seq data are available at the NCBI GEO (accession GSE288858). All other data are available as source data in the Supporting Data Values file.

## Author contributions

SS conceived the project. SS, OP, and A Sehgal designed experiments. OP, KF, LJA, MT, A Shetty, YJC, and SS performed experiments and collected data. SS, OP, AC, A Shetty, LJA, JPG, YJC, and TGB analyzed data. JPG and GG were involved in the analysis and interpretation of the transcriptomic data. OP, SS, and TGB wrote the original draft, and A Sehgal, TGB, JPG, YJC, and GG helped with revisions. SS supervised all research activities.

## Funding support

This work is the result of NIH funding, in whole or in part, and is subject to the NIH Public Access Policy. Through acceptance of this federal funding, the NIH has been given a right to make the work publicly available in PubMed Central.

- National Heart, Lung, and Blood Institute (NHLBI) R01HL155934-01A1 to SS.
- NHLBI R01HL147472 to SS.
- RCA grant from the Raine Medical Research Foundation to YJC.
- A Sehgal is an HHMI investigator.

## Supplementary Material

Supplemental data

Supporting data values

## Version 1 11/17/2025

Electronic publication
