# Supplementary material for: Effect of external cues on clock-driven protection from influenza A infection
Source: J Clin Invest. 2025 Nov 17;135(22):e193133. doi: 10.1172/JCI193133 (PMC12618070; doi:10.1172/JCI193133)
Supplement: Supplemental data [file jci-135-193133-s081.pdf]

Effect of external cues on clock-driven protection from Influenza A infection

Supplementary Figures

Table *S*1

Score	Description
1	Percolated fur but no detectable behavior differences from untreated control mice
2	Mice with percolated fur and a huddle reflex but respond to stimuli (such as a tap on their cage) appropriately and are just as active upon handling as untreated control mice
3	Exhibit a slower response to a tap on the cage and that were passive or docile when handled but still curious when alone in a new setting
4	Exhibit lack of curiosity and little response to stimuli and that appear quite immobile.
5	Exhibit labored breathing and are unable or slow to right themselves after being rolled onto their backs (moribund)
6	Dead mouse.

Table S 2

Criteria	Score
Peri-bronchial infiltrate	0-3
Peri-vascular infiltrate	0-3
Alveolar infiltrate	0-2
Epithelial injury	0-2

Supplementary Figure 1

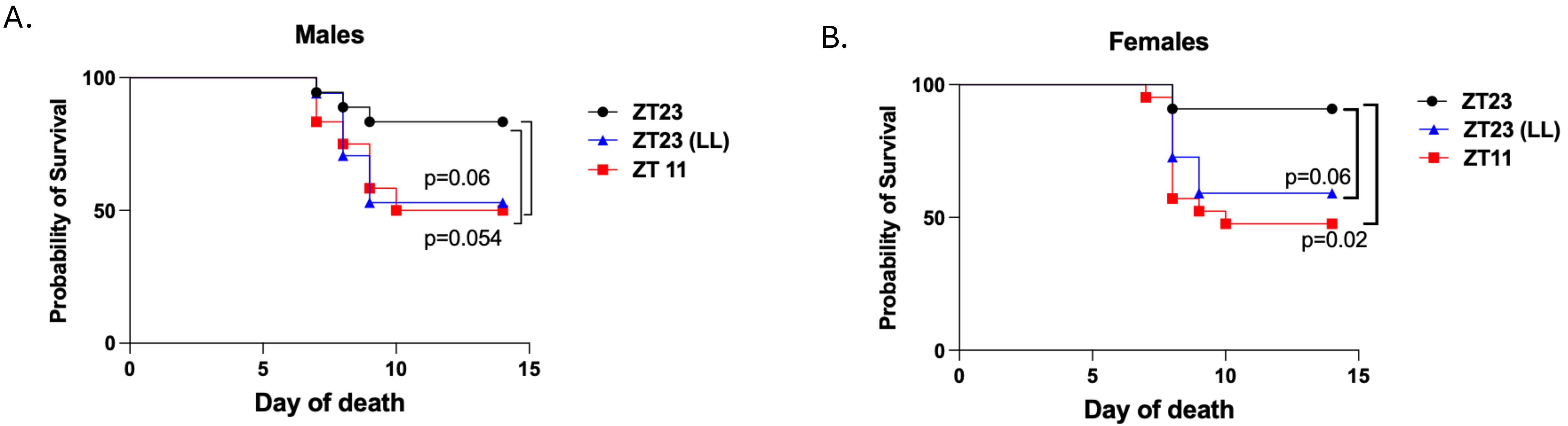

Supplementary Figure 1: Sex-stratified data for mortality following constant light exposure. (A) Males (n=12-18/group) and (B) Females (n=11-21/group) p-values as listed by Mantel-Cox Log-rank test. Summary of 3 or more independent experiments.

Supplementary Figure 2

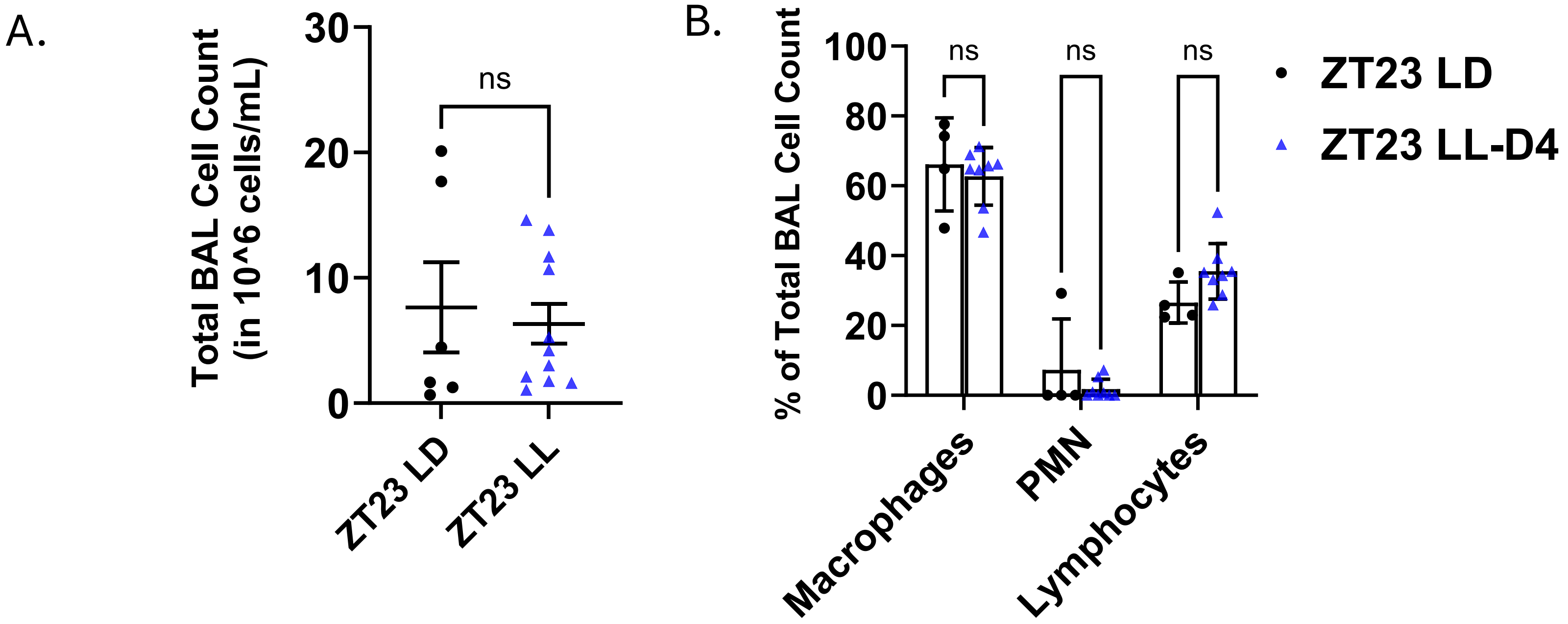

Supplementary Figure 2: Bronchoalveolar Lavage in naïve mice exposed to 4 days of constant light. (A) Total BAL count in ZT23 and ZT23(LL) groups in naïve mice. (B) BAL differential for the above data.

Supplementary Figure 3

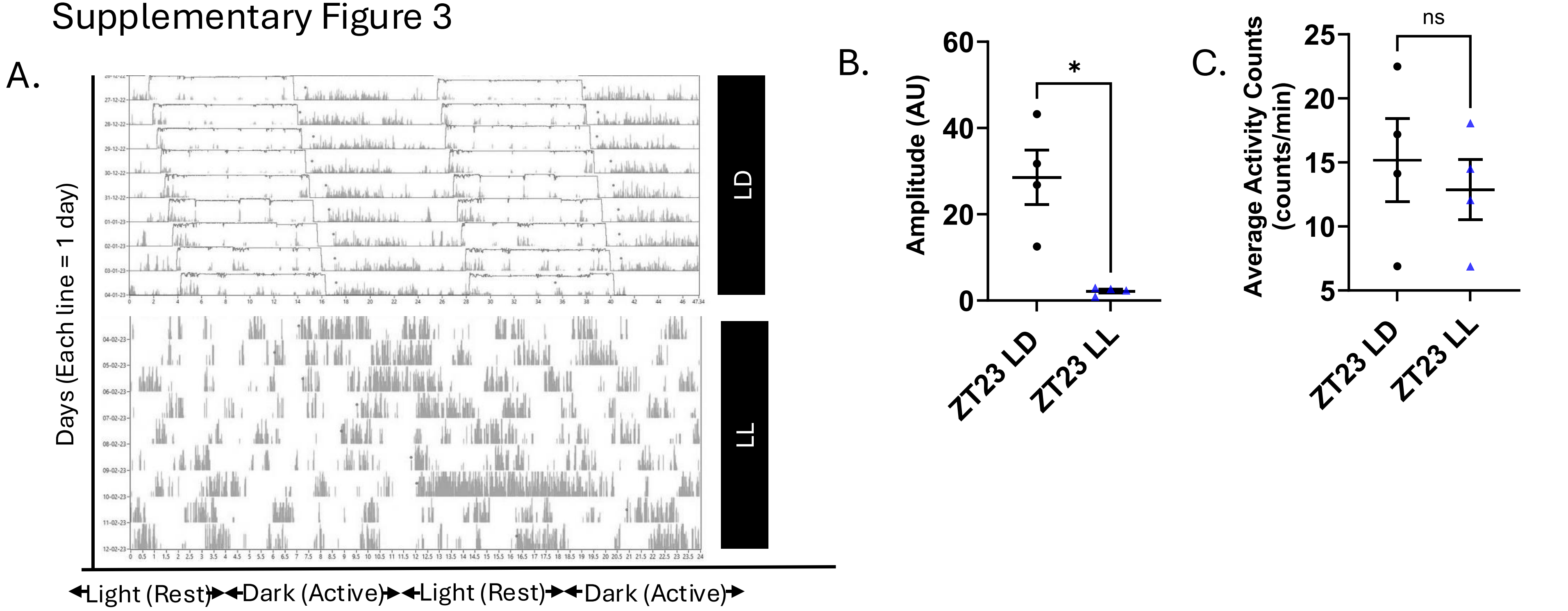

Supplementary Figure 3: (A) Locomotor activity for naïve mice in LD cycling or under constant light. (A) Representative Actogram for LD and LL naïve mice. LD data is double-plotted to clearly highlight the onset and offset of activity. LL data is across 24 hrs. (n=4). Characteristics of locomotor activity rhythms – (B) amplitude and (C) total activity count. *p<0.05 by t-test with Welch's correction.

Supplementary Figure 4

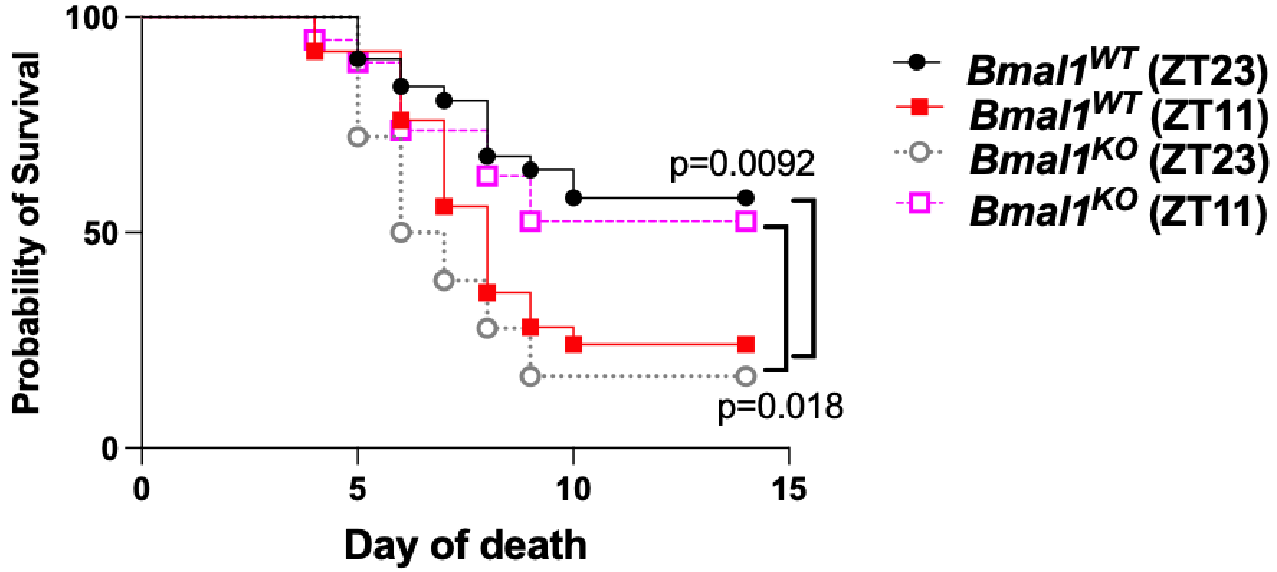

Supplementary Figure 4: Mortality from influenza infection in mice with global *Bmal1* deletion – *Bmal1*$^{fl/fl}$ *ERT2Cre*$^{+/-}$*(*or clock disruption in LD conditions). Survival (n = 19–31 per group, log-rank test). All data were pooled from three independent experiments. ns= non-significant.

Supplementary Figure 5

A.

UMAP from Sn-RNAseq (day 8 p.i.)

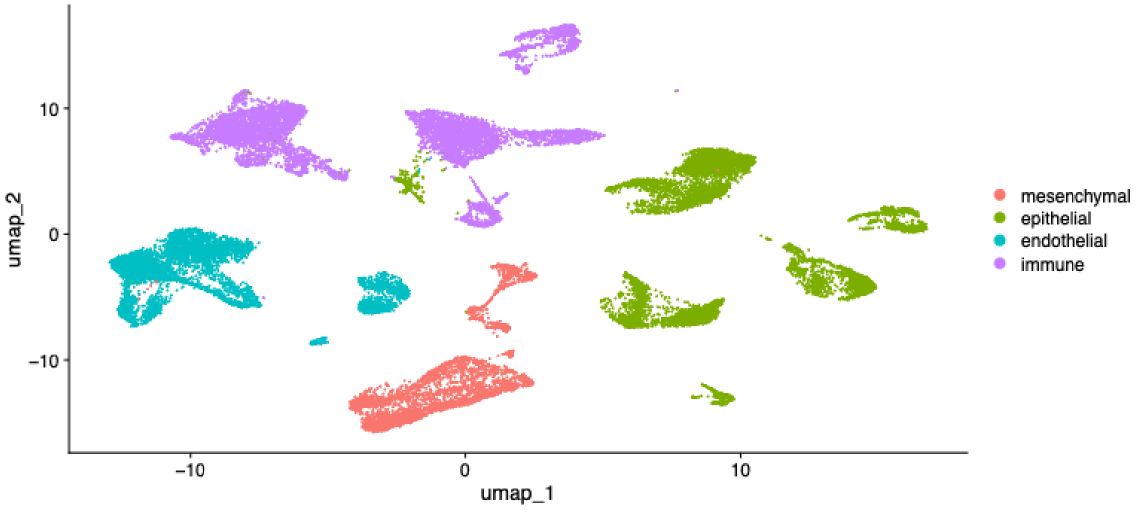

B.

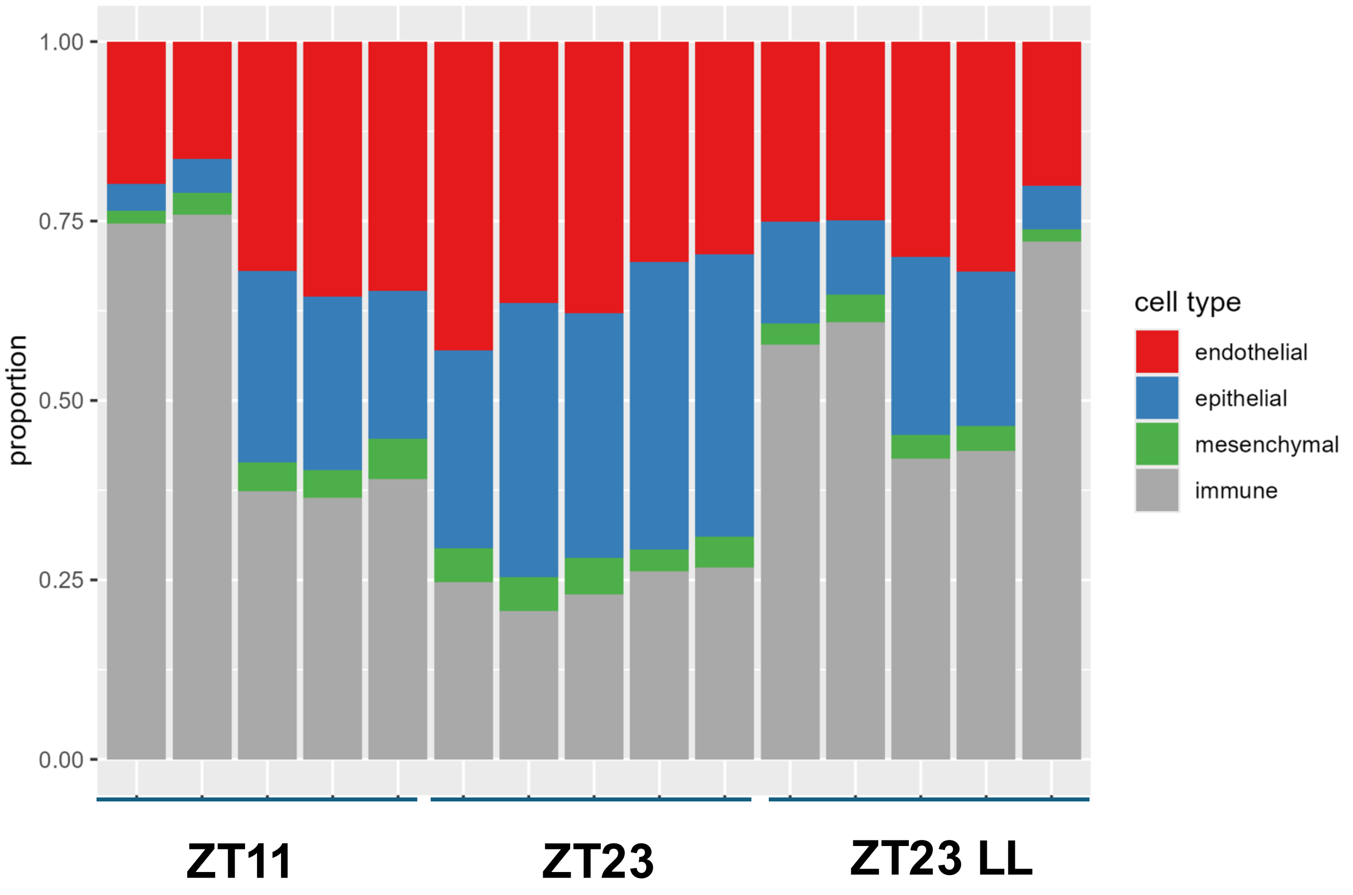

Supplemental Figure 5: (A) UMAP from Sn-RNAseq revealing cells from all four compartments of the lungs on day 8 p.i. (B) Cellular composition of the samples used for RNA-seq using a deconvolution algorithm and based on Sn-RNAseq of the whole lungs harvested from WT mice on day 8 following influenza infection.

Supplementary Figure 6

A

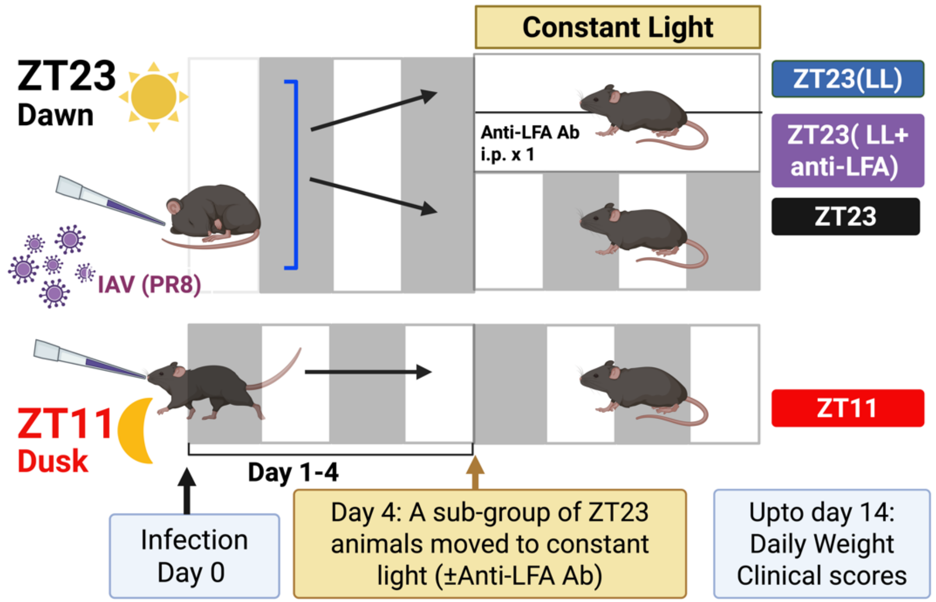

B

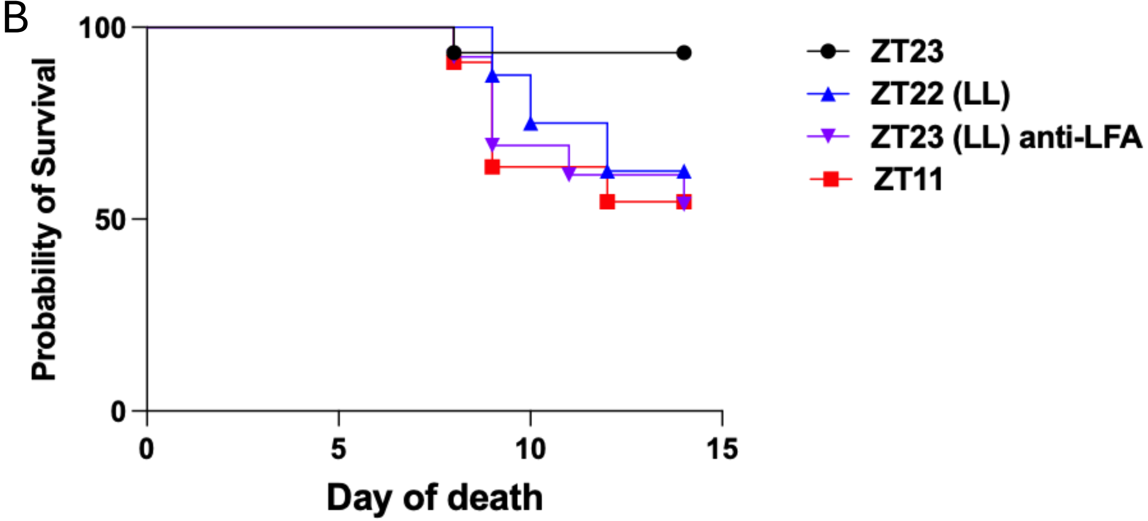

Supplemental Figure 6: Effect of blocking leukocyte migration on the loss of protection in constant light following influenza infection. (A) Experimental design. (B) Survival (n=8-15, log-rank test). All data were pooled from 3 independent experiments.
